# Mental simulation of colour properties during language comprehension: influence of context and comprehension stages

**DOI:** 10.1007/s10339-024-01201-4

**Published:** 2024-06-08

**Authors:** Donglin Liu, Lijuan Wang, Ying Han

**Affiliations:** 1 School of Psychology, Northeast Normal University, No. 5268 Renmin Street, Changchun, 130024 China; 2 Psychometrics and Quantitative Psychology, Fordham University, Bronx, NY 10458 USA

**Keywords:** Mental simulation, Colour, Language comprehension, Sentence-picture verification task, Comprehension stages, Embodied cognition

## Abstract

Many studies have shown that mental simulation may occur during language comprehension. Supporting evidence is derived from the matching effects in the sentence-picture verification (SPV) task often used to assess mental simulations of object properties, such as size, orientation, and shape. However, mixed results have been obtained regarding object colour, with researchers reporting matching or mismatching effects. This study investigated the impact of colour information clarity within sentences on the process of mental simulation during language comprehension. Employing the SPV task and using novel objects, we examined whether there is a mental simulation of colour after excluding typical/atypical colour bias and how varying levels of colour information clarity in sentences influence the emergence of matching effects at different stages of comprehension. To address these issues, we conducted two experiments. In Experiment 1, the participants read normal sentences and subsequently engaged in picture verification with a novel object after a 500 ms delay. In Experiment 2, the participants encountered sentences containing both clear and unclear colour information and, after either a 0 ms or 1500 ms interval, completed picture verification tasks with a novel object. Null effects were found in the 500 ms condition for normal sentences and the 0 ms condition for unclear colour information sentences. A mismatching effect appeared in the 0 ms condition after clear colour information sentences, and a matching effect appeared in the 1500 ms condition for all sentences. The results indicated that after excluding colour bias, the participants still formed mental simulations of colour during language comprehension. Our results also indicated that ongoing colour simulation with time pressure impacted the participant responses. The participants ignored unclear colour information under time pressure, but without time pressure, they constructed simulations that were as detailed as possible, regardless of whether the implicit colour information in the sentence was clear.

**Supplementary Information:**

The online version contains supplementary material available at 10.1007/s10339-024-01201-4.

## Introduction

Theories of grounded cognition suggest that people comprehend language through mental simulations grounded in the sensorimotor system (Barsalou 1999, 2008). Researchers have argued that the mental simulation of concepts during language comprehension partially reactivates the neural networks engaged during previous experiences (Connell 2007; de Koning et al. 2017). In other words, mental simulation involves the re-enactment of acquired experiences from details stored in sensorimotor areas. Based on this view, Barsalou (1999) suggested that comprehenders who understand the sentence “the cup on Anna’s desk is blue” would construct simulations of visual properties such as the individual desk, cup and even the colour of the cup.

Stanfield and Zwaan (2001) first investigated this hypothesis through a creative application of the sentence-picture verification (SPV) task. The SPV task first vaguely describes an object property (e.g., the orientation of a pencil) in a sentence (“John put the pencil in the cup”; implied vertical orientation of the pencil). Then, a picture with an object is presented (vertical/horizontal pencil or a filler item) to the participants, and they are asked to decide whether the object was mentioned in the previous sentence as quickly and accurately as possible. The results revealed a matching effect; the participants responded faster when the orientation of the presented object matched the orientation implied in the previous sentence, supporting the involvement of sensorimotor areas in cognitive processing. Subsequently, the SPV task has been used to investigate mental simulations of various visual properties, such as shape (e.g., Dijkstra et al. 2004; Kaup et al. 2007; Madden and Zwaan 2006; Zwaan et al. 2002), orientation (e.g., Pecher et al. 2009; Stanfield and Zwaan 2001; Wassenburg and Zwaan 2010), spatial iconicity (e.g., Zwaan and Yaxley 2003), and visibility (e.g., Yaxley and Zwaan 2007). The matching effect has been verified in these studies, except in studies on the colour of objects.

Regarding mental simulations of colour during language comprehension, mixed results have been reported. Connell (2007) adopted the SPV task to explore how the participants represent implied colour information during sentence comprehension. The results showed that the participants responded significantly faster when the presented picture did not match the previous sentence (i.e., the *mismatching condition*). Zwaan and Pecher (2012) attempted to verify the results of the study with the same sentence-picture materials but found an advantage of the matching condition. Recently, empirical evidence has supported an advantage of the matching condition (i.e., a matching effect) regarding object colour (de Koning et al. 2017; Hoeben Mannaert et al. 2017). Even in the broader context of discourse, a colour matching effect has been observed (Hoeben Mannaert et al. 2020).

Some researchers have proposed exploring the inconsistency above at the processing stage. In most studies, the pictures that needed to be verified were presented 500 ms after the participants read sentences. Therefore, Zhang and Lu (2013) suggested that the match/mismatching advantage might be related to different stages of language processing during comprehension. They proposed that the formation of mental simulation is a dynamic process that occurs throughout language comprehension. This means that although the pictures in most previous studies were presented 500 ms after the participants read sentences (Connell 2007; de Koning et al. 2017; Zwaan and Pecher 2012), the formation time of colour simulation varied among studies due to differences in experimental controls. Some participants formed colour simulations before 500 ms (e.g., de Koning et al. 2017; Zwaan and Pecher 2012), which yielded a matching effect, while others formed colour simulations after 500 ms (e.g., Connell 2007), which yielded a mismatching effect. In other words, the inconsistent results of previous studies may be due to presenting the pictures following the sentences just before or after the formation of mental simulations, thus leading to interference or facilitation. Accordingly, in their experiments, Zhang and Lu (2013) presented pictures at two different time points (0 ms versus 1500 ms) after the participants read sentences. The results displayed a mismatching effect at the early stage of comprehension (0 ms) and a matching effect at the late stage (1500 ms). Zhang and Lu argued that this is because, under the matching condition, the sentence comprehension task interferes with the picture verification task at the early stage but promotes the picture verification task at the late stage. When pictures are presented at the early stages of comprehension, the comprehenders may need to deal with both sentence comprehension and picture verification tasks simultaneously. This means that while the comprehender processes the perceptual information contained in the sentences, they also need to process the perceptual information of the pictures. Thus, two tasks may compete for the common cognitive resources of mental simulation, resulting in conflicts (Pashler 1994; Zhang and Lu 2013). Comprehenders may need to consume additional cognitive resources to suppress the interference caused by conflicts (Bai et al. 2021; Ye and Zhou 2009), resulting in reaction time extension and mismatching effects. When the pictures are presented at the late stage, the processing of sentence information by the comprehender has already finished. Therefore, mental simulation based on sentence information was able to promote picture verification under the matching condition, and hence, the matching effect occurred. Based on the experimental results, Zhang and Lu proposed a possible inflection point (e.g., 500 ms) in the formation of mental simulations during language comprehension. Other researchers have reported a similar time course of language comprehension (e.g.,Bai et al. 2021; Hasson and Glucksberg 2006; Kaup et al. 2006). For example, Bai et al. (2021) found that the object attributes (such as colour and shape) implied by a sentence were activated in the early stage of comprehension (0 ms), began to integrate in the intermediate stage (750 ms), and were completely integrated in the late stage (1500 ms). This hypothesis may explain why Zwaan and Pecher (2012) used the same exact experimental materials to replicate Connell’s (2007) study but obtained the opposite result. This outcome may have been caused by differences in individual experiences of object colour or the experimental process, leading to inconsistent durations of mental simulation formation, which manifested as a matching advantage or a mismatching advantage when the objects appeared at 500 ms. For example, for the object “steak”, people who cook frequently may be more prone to imagine a rare (red) steak, while those who cook less may be more likely to imagine a well-done (brown) steak. However, Zhang and Lu (2013) did not conduct an experiment to confirm this theory.

Other researchers have suggested that the contradictory conclusions may be explained by the clarity of the colour information implied by the sentence. Li et al. (2016) examined the effect of the clarity of colour information implied by sentences and found that the participants responded significantly faster under the matching condition after reading sentences with clear colour information. However, a null effect was found after reading sentences with less clear colour information, indicating greater contributions of clear colour information than unclear colour information to the formation of mental simulations. In their study, the authors designed two types of sentences that corresponded to differences in the clarity of colour information. The shallow sentences clearly implied the colour of the mentioned object. The deep sentences did not clearly imply the colour of the mentioned object. Because the matching effect was found under the clear colour information condition, Li et al. suggested that when a sentence implies clear colour information (e.g., “There is a mouldy banana on the floor”), the participants activate the corresponding colour perception representation (brown) based on the contextual information. However, because a null effect was found under the unclear colour information condition, they speculated that when a sentence implies unclear colour information (e.g., “There is a banana on the floor”), the colour representation (brown) is not activated. However, we propose another explanation for the null effect. Similar to the inconsistent results of previous studies, this outcome may be due to the interference of colour bias, with the typical/atypical colour (i.e., yellow vs. brown bananas) causing confusion regarding the independent variable. Our research aimed to investigate this issue.

In fact, the mental simulation of sentences during comprehension may be affected by both contextual factors and processing stages. Based on previous studies (Zhang and Lu 2013; Li et al. 2016), both variables may provide possible explanations for the formation of mental simulations; the factors are not contradictory. For instance, the sentence “*John bites a delicious tomato*” implies clearer information of a ripe tomato than the sentence “*John eats a tomato*”. Because ripe tomatoes are more strongly associated with red, red is more accessible in the first sentence. For comprehenders, it is much easier to form a specific simulation of a “*red tomato*” based on the former sentence. Thus, after reading sentences with clear colour information, people will probably form a specific mental simulation faster than they will after reading sentences with unclear colour information. Therefore, the contradictory conclusions of previous studies probably occurred because of the timing of the presented picture (500 ms), which occurred just before or after the formation of the mental simulation. That is, the participants who read sentences with clearer colour information formed a mental simulation before 500 ms, which facilitated performance on the picture verification task under the matching condition; thus, a matching effect occurred. However, the participants who read sentences with less clear colour information did not form a mental simulation before 500 ms and engaged the colour representation system required for the picture verification task under the matching condition, leading to a mismatching effect. Recently, using the SPV paradigm, researchers identified an intermediate stage of mental simulation formation between the early (0 ms) and late (1500 ms) stages after reading sentences (Bai et al. 2021), indicating that the formation of mental simulation is an ongoing process. Thus, it can be speculated that the formation of a mental simulation detectable by the matching or mismatching effect may be affected not only by the comprehension stage or the clarity of colour information but also by their interaction.

Finally, reviewing the previous experimental items, as mentioned above, we suggest that inconsistencies may be due to the interference of colour bias, i.e., typical and atypical colours of objects. In daily life, people frequently encounter familiar objects with typical colours (e.g., yellow bananas); thus, we are far more familiar with the typical colours of objects than with atypical colours (e.g., green bananas). As Engelen et al. (2011) mentioned, “Although language affords the description and simulation of unfamiliar situations, possessing a rich network of experiential traces should facilitate the rapid activation of a trace that is appropriate in a given linguistic context” (p. 661). In other words, the typical colour of an object will always activate the representation faster than an atypical colour in the equivalent context. Several studies have confirmed that the typical colour of objects is automatically activated and interferes with the processing of an atypical colour during sentence comprehension (e.g., Bramão et al. 2010, 2011; Connell and Lynott 2009; Li and Shang 2017; Tanaka and Presnell 1999). Therefore, we infer that because the object items generally used in previous studies have a typical colour, when the participants viewed objects with an atypical colour during picture verification tasks, they probably automatically activated the typical colour representations, leading to biased results. That is, interference may have introduced confounding factors that then affected the validity of results in previous experiments.

In summary, the first goal of this study is to investigate whether matching/mismatching effects occur after excluding typical/atypical colour biases. We created novel objects that were categorized into two states in our experiments, with each state corresponding to one colour. For example, the novel object called “*WAN*” (in Chinese, *萖*) was classified into two states—growing and ripe. The *growing WAN* was purple, whereas the *ripe WAN* was pink. To ensure the same level of familiarity with the colours of objects, the participants learned the two colours through a simultaneous presentation of the objects on one screen. Since the participants had never been exposed to these items before, they did not have typical/atypical colour biases due to differences with prior experience. According to James and Gauthier (2003, 2004), individuals can form colour concepts for unfamiliar objects through training. These unique stimuli could also eliminate extraneous variables that may interfere with comprehension, such as shape (for more explanation, see Hoeben Mannaert et al. 2017). The specific methods used are described in the “Procedure” section.

The second goal of the study was to investigate how implied colour information affects the formation of mental simulations at different stages of comprehension after excluding interference due to experimental materials. As considered above, the formation of mental simulations during sentence comprehension may be affected by the joint effects of comprehension stages and clarity of colour information. Therefore, the interaction effect of the two factors on mental simulation warrants further study. To our knowledge, no previous studies have jointly considered these two factors when examining mental simulation during language comprehension. Thus, we conducted two experiments.

In Experiment 1, we employed novel objects to verify whether mental simulation of an object with an implied colour occurs during sentence comprehension. Given previous studies, we predicted a matching advantage. In Experiment 2, we presented pictures at different stages of comprehension after reading sentences to examine the mechanism underlying mental simulation with varying clarity of colour information. With sentences that implied clear colour information, we expected a mismatching effect at the early stage of comprehension and a strong matching effect at the late stage. In contrast, with sentences that implied unclear colour information, we anticipated a null effect at the early stage and a matching advantage at the late stage. It is possible that at the early stage of comprehension, the comprehension task would interfere with performance on the picture verification task, supporting the hypothesis that the same neural subsystems support conceptual perception and mental simulation at the early stage of language comprehension. At the late stage of comprehension, comprehenders have had sufficient time to process the colour information implied by sentences and thus construct a mental simulation as comprehensively as possible.

## Experiment 1

### Methods

#### Participants

Thirty native Chinese speaking undergraduate students (14 males and 16 females) from Northeast Normal University participated in the experiment (age: *M* = 20.46 years old; *SD* = 1.70 years). The post hoc test showed that the power of test (1—β) = 0.754 when the significance level α ≤ 0.05 and the main effect d_z_ = 0.5. The participants who self-reported having normal colour perception and visual acuity or corrected visual acuity above 1.0 were recruited. No participant had reported colour blindness, colour weakness, or learning disabilities. No participants were involved in any pretests. The participants volunteered to join the experiment and were given a gift after finishing the experiment.

#### Materials

**Fig. 1:**
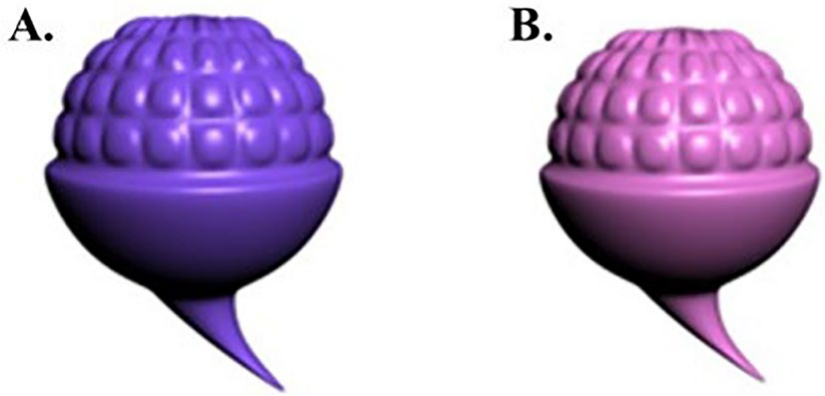
Novel object *WAN* (萖): (**A**) a *growing* (purple) *WAN*; (**B**) a *ripe* (pink) *WAN*

Fifteen novel objects were created for the experiment. Each object could exist in two different states, and each state corresponded to one colour. Thus, each object item could yield two pictures. For example, for the object “*WAN*” (*萖*), purple indicates the growing state, and pink indicates the ripe state (see Fig. 1). All objects were produced by using *Autodesk 3ds Max 2012*. The picture resolution was 3000 × 2250, both the horizontal and vertical resolutions were 96 dpi, and the size of the picture was less than 200 k. The two pictures in a pair differed only in colour to ensure that neither shape nor size was a confounding variable. Each state corresponded to one sentence, such that each item was associated with two pictures and two sentences. In addition, to prevent the participants from guessing the experimental intent or purposefully using a strategy in the picture verification task, we added sentence-word filler items to disrupt the participants. The addition of filler words could effectively prevent the participants from guessing the experimental intent or using strategies. For example, if the participants were present with only objects after reading sentences, they might adopt strategies such as consciously imagining the objects mentioned in the sentences after reading the sentences to speed up picture verification task performance. Thus, after the participants read filler sentences, filler words were presented to the participants in a random order, and the participants were asked to determine whether the words appeared in the previous sentence.

We presented the stimuli in a random order to control for the possible order effects of the experimental items and conditions. The experiment included 60 experimental sentences and 60 experimental pictures (30 sentence-picture pairs in the matching condition and 30 sentence-picture pairs in the mismatching condition; all required a “yes” response; see Table 1 for an example) and 60 pairs of filler items (all required a “no” response). Furthermore, we added 60 pairs of sentence-word filler items (60 words that corresponded to 60 filler sentences: 30 words appeared in the previous sentences and required a “yes” response; 30 words required a “no” response).

**Table 1:** Examples of experimental sentences and objects

Sentence	Matching status	
	Matching	Mismatching
John spit out the unpalatable *XUAN* (约翰吐出了难吃的*萱*) (implies a *raw XUAN*)		
John took a bite of the delicious *XUAN* (约翰咬了口好吃的*萱*) (implies a *ripe XUAN*)		

The examples provided here are shown in English, but the study used Chinese sentences. Therefore, the translation may not be exact

All experimental materials were evaluated in the pretest by 30 different participants who did not participate in the actual experiment (14 males, 16 females, *M* = 25.47 years old, *SD* = 1.65 years). These participants evaluated three aspects of the materials: the degree of novelty of the objects and names (rated on a 5-point scale, 1 not novel and 5 very novel; the lowest rating was higher than 4 points), whether the name of the object suggested a certain colour (yes or no; none of the object names were associated with colour), and understanding of the state of the novel objects and corresponding sentences (rated on a 5-point scale, 1 not understanding and 5 very understanding; the average score was higher than 4 points). For more specific information concerning the pretesting of the materials test, refer to the Experiment 1 section of the supplementary materials. The experimental and filler sentences were presented in a random order.

### Design and procedure

We designed the experiment to be analysed with single-factor analysis. The independent variable was the matching status of the colour implied by the sentences and that presented in the pictures. The matching status had two levels: matching or mismatching. The dependent variable was RT. The experiment was divided into a training phase and an experimental phase. All experimental programs were created and presented in E-Prime 2.0.

*Training phase* Some studies have investigated the feasibility of learning artificially generated concepts (e.g., Gauthier et al. 2003); thus, our study used a mature training technique. Drawing from previous work (Gauthier et al. 2003; Cheung and Gauthier 2014; James and Gauthier 2003, 2004), we divided the training procedure into two stages.

**Fig. 2:**
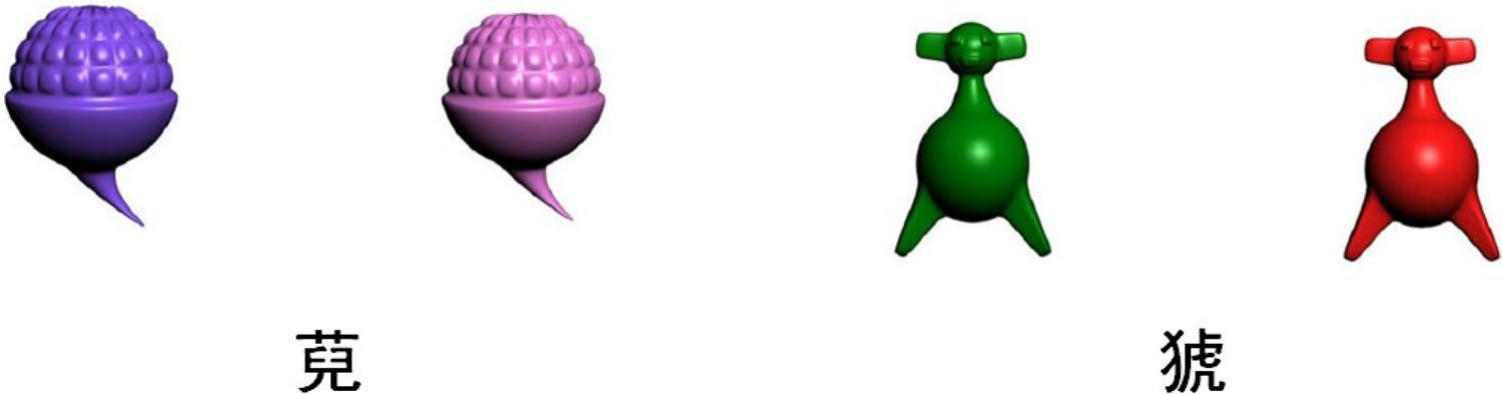
Examples of the first-stage training materials. The left panel presents two differently coloured *WANs* (萖), a novel plant-like object; the right panel presents two differently coloured *XIAOs* (猇), a novel animal-like object

During the first training stage, an object name and two colour versions of the object were presented simultaneously (see Fig. 2). The participants controlled the presentation duration. Once the participants believed that they had carefully learned information about the object, they could press the spacebar to advance to the next item. All items were presented in a random order, and the participants could repeat the learning stage at any time. After learning was complete, the participants were asked to perform complex mathematical operations for 5 min before moving on to the test phase. The aim of the test and interval was to ensure that the objects were retrieved from long-term memory rather than working memory.

During the test phase, novel objects in a certain colour or the names of novel objects appeared randomly on the screen. The participants needed to recall the novel objects and their corresponding names and colours and orally report this information to the experimenter. The experimenter recorded the participants’ scores. Those who obtained a perfect score (i.e., they perfectly mastered all the novel objects) advanced to the second training stage. Those who failed to achieve full marks repeated the first training stage to relearn the objects. After repeated learning sessions, all participants achieved full marks.

**Fig. 3:**
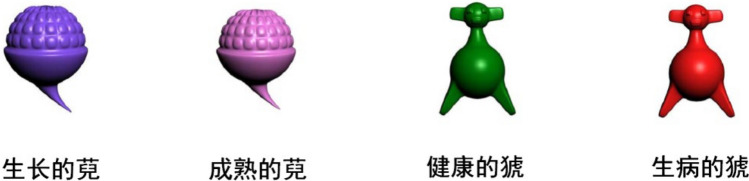
Examples of the second-stage training materials. The left panel presents two differently coloured *WANs* corresponding to two states, a growing *WAN* (生长的萖) and a ripe *WAN* (成熟的萖); the right panel presents two differently coloured *XIAOs* corresponding to two states: a healthy *XIAO* (健康的猇) and a sick *XIAO* (生病的猇)

In the second training stage, both colour versions of an object were simultaneously presented; under each version, there was a phrase indicating the state of the object (Fig. 3). The learning process was similar to that in the previous stage. After sufficiently learning the information, another 5-min session of complex mathematical operations was included before the test phase to ensure that the objects were retrieved from long-term memory rather than working memory. During the test phase, either novel objects in a certain colour or the names of the novel objects appeared randomly on the screen. The participants needed to recall the novel objects and their corresponding names, colours and states. They orally reported this information to the experimenter, and the experimenter recorded the participants’ scores. Again, those who obtained a perfect score in the test phase advanced, whereas others repeated the training stage. All participants achieved full marks through repeated learning sessions and entered the formal experimental process.

*Experimental phase* In the formal experiment, the participants were first asked to read the instructions, which asked them to read every sentence as quickly and carefully as possible, and press the spacebar after reading the sentence. They were informed that each sentence would be followed by a picture or word and that their task was to decide whether the depicted object was mentioned in the preceding sentence. The participants were also informed that various aspects of their comprehension would be tested during the experiment, including the occasional presentation of a comprehension sentence to test their comprehension of the previous sentence. These comprehension sentences were presented after the picture verification task in a random order, and the participants had to decide whether the meaning of the comprehension sentence was consistent with the sentence that they had read. The comprehension sentences usually evaluated the participants’ understanding of the preceding sentence to ensure that they had carefully read the sentence (e.g., the WAN was not yet ripe). Half of the comprehension sentences corresponded to the meaning of the previous sentence (correct response: press the “J” key), while the other sentences did not correspond to the meaning of the previous sentence (correct response: press the “F” key). After pressing the key, the participants entered the next trial. They were further asked to respond as quickly and accurately as possible because their RTs and performance accuracy would be measured. To help the participants become familiar with the experimental tasks, before the formal experiment, we performed a practice experiment that included four trials. During each trial, the participants first looked at a left-aligned vertically centred fixation cross for 1000 ms; next, a sentence was presented. After the participant pressed the spacebar to indicate they had completed the reading, another fixation cross was displayed centrally on the screen for 500 ms, followed by the presentation of a picture or word. The participants were asked to determine whether the pictured object had been mentioned in the preceding sentence (if mentioned, press the “J” key; if not, press the “F” key), and then followed a comprehension sentence, occasionally. After the participants were familiar with the experimental process, they pressed the “J” key to enter the formal experiment. The procedure of the formal experiment was consistent with that of the practice experiment. Since all objects in the experimental pictures were mentioned in the preceding experimental sentence, the correct response in the recognition judgement task was to press the “J” key. No filler pictures had been mentioned in the preceding sentence; thus, the correct response was to press the “F” key. Each participant completed a total of 60 experimental trials. The data on the filler items were not recorded. The whole experiment took approximately one hour to complete.

### Statistical analysis

First, in line with the procedure used by previous studies (e.g., Zwaan and Pecher 2012; de Koning et al. 2017), for every SPV task, the data of the participants with an accuracy of 0.80 or lower were excluded from the analyses because unusually low accuracy scores on this task are considered to not accurately reflect the cognitive processes that the task aims to evaluate. One participant was excluded because of an accuracy of less than 0.80. Second, we excluded trials in which the participants responded incorrectly. Third, following the procedure described by Connell (2007), all RTs shorter than 300 ms or longer than 3000 ms and all responses more than 2.5 standard deviations (SDs) from the mean RTs at the participant level under the relevant condition were removed. Finally, data from 29 participants (14 males, 15 females) were included in the subsequent analysis. Twenty-three trials, accounting for approximately 1.36% of the collected data, were ultimately excluded.

Using *SPSS 22.0* and *JASP 0.9.2*, we performed paired-sample *t* tests and Bayesian analyses. The dependent variable was the reaction times (RTs) of the participants to picture verification. Because we were concerned that the number of experimental trials completed by each participant was too small, following Zwaan and Pecher (2012), we conducted an analysis focusing on both the mean and median RTs. The results were analysed for two conditions: matching (the colour implied in the sentence is consistent with the colour of the object presented in the picture) and mismatching (the colour implied in the sentence is inconsistent with the colour of the object presented in the picture). We considered *p* < 0.05 to be statistically significant.

## Results

We calculated the mean and median reaction times of each condition, as Table 2 shows. Further analysis was carried out between matching and mismatching conditions to identify the posterior probabilities (BF_01_) favouring the null hypothesis. If BF_01_ is larger than 1, adding the effect to the model makes the evidence supporting the null hypothesis more compelling (Masson 2011). We found no significant differences in mean RTs (*t*(29) = 0.888, *p* = 0.382, *BF*_*01*_ = 3.533) or median RTs (*t*(29) = 1.291, *p* = 0.207, *BF*_*01*_ = 2.392) between the matching and mismatching conditions. The results indicated no significant differences in RTs between the matching and mismatching conditions when pictures were presented 500 ms after the participants comprehended the sentences.

**Table 2:** M and SD of mean and median reaction times (ms) in the two conditions (n = 29)

	Mean		Median	
Condition	M	SD	M	SD
Matching	1326.88	266.65	1316.16	258.11
mismatching	1309.97	269.85	1288.71	265.65

## Discussion

Our aim was to investigate whether there was a matching/mismatching advantage after excluding typical/atypical colour biases by eliminating the experimental material as an interference factor. Thus, as a conceptual replication study, our research presented the same picture presented at the time point (500 ms after the participants read the sentences) usually employed in previous studies, and obtained a null effect. Although the lack of significant differences seems not support some aspects of embodied cognition theory, which posits that language comprehension involves simulating sensory experiences (Barsalou 2008). However, we cannot definitively conclude that there was no mental simulation during language comprehension. The null effect may be due to the experimental material.

The clarity of the colour information implied in sentences may affect mental simulation; compared with sentences with unclear colour information, sentences with clear colour information may have allowed comprehenders to easily form the corresponding colour simulation after reading (Li et al. 2016). Since we did not control for the clarity of colour information in the sentence materials; thus, when comprehenders read sentences, variations in the clarity of colour information in sentences may have led to mental simulations occurring at different time points. Due to this mix of matching and mismatching advantages, when a picture was presented at 500 ms, a null effect was observed.

Because Experiment 1 did not control for the clarity of the colour information in the sentence materials, it was difficult to determine the exact cause of the null effect. Therefore, in Experiment 2, we controlled for the clarity of the colour information implied in sentences, dividing sentences into those that implied clear and unclear colour information; we then explored the effects of these colour-clarity conditions and comprehension stages on the mental simulation of colour.

## Experiment 2

The results of Experiment 1 showed a null effect; this null effect was likely due to the two potential causes described above. Therefore, to further explore the causative factors and internal mechanisms of mental simulation, we opted to avoid the controversial time point of 500 ms. Referring to the research of Zhang and Lu (2013), we set the picture presentation time to correspond to the early (0 ms) and late (1500 ms) stages of comprehension. Moreover, we did not control for the clarity of colour information in the sentence materials in Experiment 1. Thus, to further investigate the effect of the clarity of colour information, the sentence items were divided into clear and unclear colour information conditions.

### Methods

#### Participants

Due to the mixed factorial design and the use of the comprehension stage as the between-subjects variable, two groups of participants were needed for Experiment 2. Thus, 60 native Chinese speaking undergraduate students (27 males and 33 females) from Northeast Normal University were recruited to participate in the experiment (*M* = 20.32 years; *SD* = 1.37 years). The post hoc test showed that the power of the test (1—β) = 0.861 when the significance level was α ≤ 0.05 and the interaction effect was f = 0.40. The participants who self-reported having normal colour perception and visual acuity or corrected visual acuity above 1.0 were recruited. No participant had reported colour blindness, colour weakness, or learning disabilities. None of the participants took part in Experiment 1 or any similar experiments. All participants were given a gift after finishing the experiment.

#### Materials

A total of 15 novel objects were created based on the same parameters described in Experiment 1. One novel object was associated with 2 experimental pictures and 4 sentences (2 clear- and 2 unclear-colour information sentences, see Table 3). Before Experiment 2, the clarity of the colour information implied by the sentence materials was evaluated in a pretest with the 30 participants who participated in the evaluation of the Experiment 1 materials. The participants determined which sentences had clear/unclear colour information and evaluated the gradient of colour information. All participants successfully distinguished between the sentences with clear/unclear colour information, and the gradient between the clear/unclear colour information that the sentences implied significantly differed (rated on a 5-point scale; *t*(30) = 37.01, *p* < 0.001; *M*_high_ = 3.82, *SD*_high_ = 0.24; *M*_low_ = 1.94, *SD*_low_ = 0.17). For more specific information concerning the pretesting of the materials test, refer to the Experiment 2 section of the supplementary materials.

**Table 3:** Examples of experimental sentences and objects

Sentence		Matching status	
		Matching	Mismatching
Sentences implying clear colour information	Bonnie saw a jumping *LUO* at the pond (邦妮在池塘边看到一只蹦跳的*猡*) (implies a *live LUO*)		
	Bonnie saw a cooked *LUO* in the saucepan (邦妮在锅里看到一只煮熟的*猡*) (implies a *dead LUO*)		
Sentences implying unclear colour information Low	Bonnie saw a *LUO* at the pond (邦妮在池塘边看到一只*猡*) (implies a *live LUO*)		
	Bonnie saw a *LUO* in the saucepan (邦妮在锅里看到一只*猡*) (implies a *dead LUO*)		

The examples provided here are in English, but the study used Chinese sentences. Therefore, the translation may not be exact

#### Design and procedure

We applied a 2 matching condition (matching and mismatching) × 2 clarity of colour information (clear and unclear) × 2 comprehension stage (0 ms and 1500 ms) experimental design. The first two factors were within-subject factors, and the third factor was a between-subjects factor. The dependent variable was RT.

*Training phase* The training phase of Experiment 2 was consistent with that of Experiment 1, including two stages.

*Experimental Phase* The design and procedure of Experiment 2 were identical to those of Experiment 1, except that pictures were presented at either the early or late stage (0 or 1500 ms after the participants read the sentences, respectively).

### Statistical analysis

The data processing and analysis in Experiment 2 followed the same procedures and criteria as in Experiment 1. Two participants in the 0 ms group were excluded because their accuracy was less than 0.80. Ultimately, data from 58 participants (27 males, 31 females) were included in the subsequent analysis, and a total of 137 trials, accounting for approximately 1.968% of the collected data, were excluded. The data were submitted to a repeated measure ANOVA using *SPSS 22.0*.

The dependent variable was the RTs of the participants to picture verification. The results were analysed for three conditions: clarity of colour information (clear and unclear), matching condition (matching and mismatching), and comprehension stage (0 ms and 1500 ms). The first two factors were within-subject factors, and the third factor was a between-subjects factor. Using repeated-measures ANOVA, we analysed the interaction of the three conditions and the main effects of each condition. Furthermore, a simple effect analysis of the comprehension stage was used to explore the interaction between the clarity of colour information and the matching condition. We considered* p* < 0.05 to be statistically significant. Eta squared (*η*_*p*_^2^) was regarded as an effect-size measure.

## Results

We calculated the means and SDs of RTs for each condition, as shown in Table 4. The ANOVA results showed significant main effects of the clarity of colour information (*F*(1, 57) = 110.609, *p* < 0.001, *η*_*p*_^2^ = 0.664), comprehension stage (*F*(1, 57) = 74. 539, *p* < 0.001, *η*_*p*_^2^ = 0.571) and matching condition (*F*(1, 57) = 19.238, *p* < 0.001, *η*_*p*_^2^ = 0.256). There were significant interactions of the clarity of colour information with the comprehension stage (*F*(1, 57) = 100.891, *p* < 0.001, *η*_*p*_^2^ = 0.643) and matching condition (*F*(1, 57) = 29.633, *p* < 0.001, *η*_*p*_^2^ = 0.346). The matching condition also significantly interacted with the comprehension stage (*F*(1, 57) = 206.522, *p* < 0.001, *η*_*p*_^2^ = 0.787). The three-way interaction among the clarity of colour information, matching condition, and comprehension stage was also significant (*F*(1, 57) = 47.767, *p* < 0.001, *η*_*p*_^2^ = 0.460).

**Table 4:** M and SD of reaction times (ms) in different conditions (n = 58)

	Clear		Unclear	
Comprehension stage	Matching	Mismatching	Matching	Mismatching
Early (0 ms)	1761.457 (190.78)	1543.020 (172.54)	1916.458 (211.79)	1907.118 (195.53)
Late (1500 ms)	1294.689 (161.55)	1521.057 (185.28)	1313.075 (145.21)	1514.603 (160.67)

The simple effects analysis results showed that the interaction between the clarity of colour information and matching condition was significant at the early stage (0 ms) of comprehension (*F*(1, 27) = 48.570, *p* < 0.001, *η*_*p*_^2^ = 0.643). Further analysis showed that after the participants read sentences implying clear colour information, the RTs under the mismatching condition were significantly shorter than those under the matching condition (*F*(1, 27) = 111.81, *p* < 0.001, *η*_*p*_^2^ = 0.805). However, we found no significant differences in the RTs between the matching and mismatching conditions after the participants read sentences implying unclear colour information (*F*(1, 27) = 0.21, *p* = 0.646, *η*_*p*_^2^ = 0.008). In addition, under both the matching and mismatching conditions, the RTs were significantly shorter after the participants read sentences implying clear colour information than after they read sentences implying unclear colour information (*F*_matching_ (1, 27) = 55.01, *p*_matching_ < 0.001, *η*_*p*_^2^ = 0.671; *F*_mismatching_(1, 27) = 255.49, *p*_mismatching_ < 0.001, *η*_*p*_^2^ = 0.904).

However, at the late stage (1500 ms), the interaction between the clarity of colour information and matching condition was not significant (*F*(1, 29) = 2.16, *p* = 0.153, *η*_*p*_^2^ = 0.069). Further statistical analysis showed a significant main effect of matching condition (*F*(1, 29) = 252.32, *p* < 0.001, *η*_*p*_^2^ = 0.897). Regardless of the clarity of colour information, the RTs under the matching condition were always significantly shorter than those under the mismatching condition (*F*_clear_ (1, 29) = 205.73, *p*_clear_ < 0.001, *η*_*p*_^2^ = 0.876; *F*_unclear_ (1, 29) = 158.24, *p*_unclear_ < 0.001, *η*_*p*_^2^ = 0.845). However, the main effect of the clarity of colour information was not significant (*F*(1, 29) = 0.16, *p* = 0.696, *η*_*p*_^2^ = 0.005). Regardless of the matching condition, there was no significant difference between the RTs in the clear colour information condition and the RTs in the unclear colour information condition (*F*_matching_(1, 29) = 1.64, *p*_matching_ = 0.211, *η*_*p*_^2^ = 0.054; *F*_mismatching_(1, 29) = 0.11, *p*_mismatching_ = 0.747, *η*_*p*_^2^ = 0.004).

## Discussion

The purpose of Experiment 2 was to examine how the processing of colour during mental simulation was influenced by the clarity of colour information during language comprehension. Our results showed that during the early stage (0 ms), a mismatching advantage was observed for sentences implying clear colour information, and a null effect was noted for sentences implying unclear colour information. However, during the late stage (1500 ms), a matching advantage was detected; RTs under the matching condition were always shorter than those under the mismatching condition regardless of the clarity of colour information. Due to the mixed results obtained, mental simulation of the implied colour during language comprehension has a more complicated internal mechanism. These issues are further discussed in detail in the *General Discussion*.

### General discussion

The present study aimed to exclude the interference factors of the materials and control for the possible influence of additional variables, such as individual experience and object shape, on the experimental results, unlike previous studies. Thus, two experiments were performed to explore the mechanisms of the mental simulation of colour during sentence comprehension. The data supported the hypothesis that people construct mental simulations of the colour implied by sentences during comprehension and that the formation of mental simulations during sentence comprehension is an ongoing process. The results showed that after the participants read sentences implying clear colour information, presenting a picture at the early stage of comprehension (0 ms) led to the interference of the sentence comprehension task with the picture verification task, resulting in a mismatching advantage. In the middle stage of comprehension (e.g., 500 ms), there seems to be an inflection point of the formation of mental simulation, leading to a null effect. In contrast, if the picture was presented at the later stage of comprehension (1500 ms), the sentence comprehension task facilitated performance on the picture verification task, resulting in a matching advantage. However, when the participants read sentences implying unclear colour information, the matching advantage appeared only when the picture was presented at the late stage of comprehension.

Contrary to previous investigations, we did not find a significant effect in Experiment 1. Although we obtained a null effect, this does not mean that there was no mental simulation of colour during language comprehension. Some researchers have suggested that there is an intermediate stage of language comprehension or an inflection point in simulation formation (e.g., Bai et al. 2021; Hasson and Glucksberg 2006; Kaup et al. 2006; Zhang and Lu 2013). In other words, the null effect may be due to the timing (500 ms) of picture presentation after reading the sentences in Experiment 1. This time point may be at the intermediate stage of mental simulation formation and the inflection point of simulation formation. The picture presentation may have occurred before or after the completion of the mental simulation. This may lead to facilitation and interference, respectively, with performance on the picture verification task. This mix of matching and mismatching advantages may have led to a null effect. Some researchers have suggested that the knowledge and experience of objects can affect the formation speed of mental simulation; the richer the experience of objects is, the faster an individual forms mental simulations (Engelen et al. 2011). However, we used novel objects that are less familiar than common objects in daily life, limiting the participants’ experience with these objects, which may have caused mental simulation to occur later. Since our study used materials different from those of previous studies, it is difficult to determine whether the cause of the null effect was that the participants were unfamiliar with experimental materials resulting in simulations not being formed by 500 ms or that the inflection point was located at the intermediate stage of comprehension due to the variation in the clarity of colour information implied by the sentence materials. To circumvent the inflection point of mental simulations and thus address our second goal, in Experiment 2, we presented picture items at the early/late stage of comprehension and used sentence items with clear/unclear colour information to investigate how context influences the mental simulation of implied colour.

The results of Experiment 2 indicated a complex mechanism underlying mental simulation during language comprehension. Similar to Zhang and Lu (2013), we found a mismatching effect at the early stage and a matching effect at the late stage. The mismatching effect at the early stage of our findings seemed to provide some supporting explanations for Connell (2007). According to embodied theory, when comprehenders attempt to understand sentences, the neural systems that respond to the visual perception of novel objects are engaged, and the same neural systems are involved in the subsequent picture verification task under the matching condition (Connell 2007). For example, the sentence “William is watering the thriving *WAN*” activated the visual experience trace for a purple (growing) *WAN*, and a picture of a purple *WAN* then activated the visual experience trace of the purple *WAN*. Thus, when asked to determine information during the early stage under the matching condition, the participants could not immediately recruit the neural systems to support performance on the picture verification task since these neural systems were occupied with processing the purple *WAN* from the sentence comprehension task. This competition caused disruption in the performance of the picture verification task and led to longer RTs. When asked to determine information during the early stage under the mismatching condition, sentence comprehension task performance and picture verification task performance did not compete for the same neural systems, resulting in shorter RTs than those under the matching condition; thus, a mismatching effect occurred. However, at the late stage, the participants had sufficient time to process the comprehension task. Hence, the neural system that was activated during the sentence comprehension task was free to be recruited for (and facilitate) performance on the match picture verification task; thus, a matching effect was obtained. Therefore, in our research, the mismatching and matching effects are not contradictory. Whether it is the occurrence of matching or mismatching facilitation, both support the hypothesis of the embodied theory that colour simulation formed during language comprehension engages the same sensorimotor system as colour perception (Barsalou 1999). However, in the early stages of sentence implied unclear colour information conditions, the occurrence of a null effect seems to indicate that participants ignored the deeper hidden colour information during comprehension. Interestingly, our study revealed a greater matching advantage during the late stage of comprehension than previous research regardless of the clarity of colour information (clear colour information condition: 226 ms, [1295 vs. 1521 ms]; unclear colour information condition: 201 ms, [1313 vs. 1514 ms]; Zwaan and Pecher (2012), 56–157 ms, [1232 vs. 1288 ms]). Under the mismatching condition in our study, the participants apparently experienced more interference from the sentence comprehension task during the performance of the picture verification task, most likely because the novel objects had a single colour—unlike the normal objects—and the participants were more likely to form mental simulations of the objects with specific colour characteristics than to form mental simulations of the normal objects, resulting in a greater conflict between these two tasks. In summary, our findings reveal mental simulation during language comprehension and support the view of embodied theory, which posits that concepts are partially stored in the neural systems of perceptual experiences.

Our findings are consistent with those of other studies, and the interference and facilitation between mental imagery and visual perception in our data and others (Kosslyn 1994; Lloyd-Jones and Vernon 2003) align with the mismatching effects found for other object aspects, such as motion. For example, Kaschak et al. (2005) asked participants to listen to a sentence that described a directional movement (e.g., “The car approached you”). Concurrently, they viewed a dynamic black-and-white stimulus that produced the perception of movement either in the same direction as the action specified in the sentence (i.e., towards the participants) or in the opposite direction (i.e., away from the participants). The participants responded faster when the sentence described a direction opposite to the present visual motor stimulus, suggesting that the processing mechanisms recruited to form simulations during language comprehension can also be involved in visual perception, like a motion aftereffect that occurs in language comprehension (Pavan and Baggio 2013; Slivac and Flecken 2023). Recently, researchers have found that there is an adaptation aftereffect in colour similar to a motion aftereffect in the process of language comprehension (Hoeben Mannaert et al. 2020; Zheng et al. 2017). Researchers have proposed that the semantic representation of colour may share a neural substrate with colour perception (Zheng et al. 2017) and even those mental simulations of different perceptions still have a common ‘core’ neurocognitive system (Mahr 2020). Therefore, in the process of comprehension, comprehenders simulate the colour information in the sentence, which will interfere with the subsequent verification of diagrams or pictures (Connell 2007; Zhang and Lu 2013; Zheng et al. 2017). Based on the hypothesis of common neural substrate, in our study, after comprehenders read the sentence implying clear colour information and a matching picture is presented at the early stage of comprehension (0 ms), they need to process both the sentence comprehension task and the picture verification task. Thus, the two tasks will employ a common neural substrate of colour perception, which will cause mutual competition between the tasks, resulting in a longer reaction time and mismatch effect. Otherwise, when a matching picture is presented at the late stage of comprehension (1500 ms), comprehenders have sufficient time to process sentences. Thus, comprehenders have completed the integration of colour information implied before the picture verification task and formed a complete mental simulation including colour (Bai et al. 2021). This would contribute to the verification of the matching pictures, and the matching effect would occur.

Second, as mentioned above, our results support previous studies showing that there is an inflection point or intermediate stage during language comprehension (Bai et al. 2021; Hasson and Glucksberg 2006; Kaup et al. 2006; Zhang and Lu 2013). Our data indicate that mental simulation is an ongoing process and that people ignore unclear property information when they form mental simulations under time pressure; however, without time pressure, their mental simulations contained unclear property information. In Experiment 2, at the early stage of comprehension, we found no significant differences in the RTs between the matching and mismatching conditions when the participants finished the verification tasks after they read sentences with unclear colour information. This result is similar to the findings of Li et al. (2016), who reported a null effect under unclear colour information conditions. One possible explanation may be that the colour information implied by sentences with unclear colour information was buried so deeply that it was difficult for the participants to extract it in a limited time. Therefore, they ignored unclear colour information due to the time constraints and constructed a mental simulation with no specific colour; thus, they compared only identical properties between the simulation and object, resulting in a null effect. However, this does not mean that individuals completely ignore unclear colour information during comprehension because we also found a matching effect under the unclear colour condition at the late stage of comprehension. This finding probably means that when individuals have enough time to process a sentence, even if its colour information is deeply buried, they construct mental simulations as comprehensively as possible in the allotted time. Future research should address this phenomenon with other object properties.

Finally, when the participants finished the verification tasks at the early stage of comprehension, clear colour information promoted sentence comprehension performance. We found that at the early stage of comprehension, regardless of the matching condition, the participants responded faster after reading sentences with clear colour information than after reading sentences with unclear colour information. No significant difference was found in the RTs between the clear and unclear colour information at the late comprehension stage. In other words, when a sentence has clear colour information, it is easier for individuals to understand the colour information implied by the sentence, enabling them to make a faster determination within a limited time. However, colour information does not affect RTs when people have sufficient time to understand the information.

### Limitations

First, due to the limited experimental materials, each picture in Experiment 1 was repeated four times, while each picture in Experiment 2 was repeated 8 times (adding new experimental items effectively required adding a corresponding filler item, which is equivalent to adding two objects, four colours and four states, thus greatly increasing the learning load of the participants). The duplication of materials may have affected the results of the experiment. Therefore, we added word filler items to reduce the effect of repeated pictures on the participants. However, this limitation may have affected the results and should be considered and addressed in future studies.

Second, our study did not account for individual differences, such as language skills and colour perception. Although we controlled the participants’ learning performance for novel objects, only those who achieved a full score could participate in the formal experiment. However, language skills may affect not only the participants’ understanding of the description state of novel objects during the learning process but also their perceptions of implicit colour information in sentences during SPV tasks. The participants who had better language skills may have more easily obtained implicit colour information in sentences, which in turn affected the experimental results. In addition, we selected the participants who self-reported having normal colour perception. However, the difference in colour perception may lead to the difference in the recognition of objects with different colours in the picture verification task. Our study did not assess individuals’ differences in language skills and colour perception. Therefore, future studies should consider individual differences and might consider individual differences in subjects and items using mixed-effects modelling in their statistical analysis.

Finally, our study asked the participants to immediately press the button after reading the sentence. This requires the participants to control their own reading time, and individual subjective differences may have a greater impact on reading time. It may affect the measurement of the formation time of mental simulation. Future studies should control for the subjective factors among the participants via time-constrained paradigms. For example, in an SPV task, sentences are presented for a fixed time span (e.g., 1500 or 2000 ms), after which the participants are automatically advanced to an ISI or the picture verification task.

## Conclusion

In the current study, we adopted an improved experimental method involving the use of novel objects in the SPV task to minimize typical/atypical colour biases to explore mental simulation during language comprehension. In Experiment 1, a null effect was observed. In Experiment 2, a mismatching effect was observed under the clear colour information condition at the 0-ms stage, and a matching effect was observed under both the clear and unclear colour information conditions at the 1500-ms stage. These results indicate that colour information can be represented in simulations regardless of whether an object is shown in a typical (or atypical) colour. Furthermore, we surmise that regardless of whether an object has a typical (or an atypical) colour, individuals could form mental simulations based on the colour information that sentences imply.

We also concluded that the mental simulation of implied colour information during sentence comprehension was influenced by both the clarity of colour information and the comprehension stage. Previous inconsistent results may be due to the differences between the clear and unclear colour information of sentence materials, leading the commonly used presentation time (500 ms) to become an inflection point between different simulation stages. In general, we suspect that the clarity of implied object properties in a sentence affects the speed of mental simulation formation. Although processing the colour information of an object deeply buried in a sentence (i.e., unclear implied colour information) did not facilitate sentence comprehension, individuals still used unclear information to construct mental simulations as comprehensively as possible.

## Supplementary Information

Below is the link to the electronic supplementary material.

Supplementary file1 (DOCX 642 kb)
