# Supplementary material for: Mental simulation of colour properties during language comprehension: influence of context and comprehension stages
Source: Cogn Process. 2024 Jun 8;25(4):587–600. doi: 10.1007/s10339-024-01201-4 (PMC11541384; doi:10.1007/s10339-024-01201-4)
Supplement: Supplementary file 1 — Supplementary file1 (DOCX 642 kb) [file 10339_2024_1201_MOESM1_ESM.docx]

**Supplementary materials: Pre-testing data for Experiment 1**

| Name | Senences | Novel items | | | | Score (M, SD) | |
| --- | --- | --- | --- | --- | --- | --- | --- |
|  |  | Objects | | States | |  |  |
| 猣  ZONG | 强森在放牧时看到了一只猣。  Johnson saw a ZONG while grazing. | 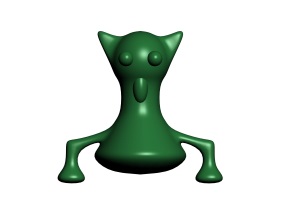 | | ZONG living in the grassland | | Name: 4.067 (0.85)  object: 4.667 (0.70)  state1: 4.100 (0.79)  state2: 4.033 (0.80)  Sentence1: 4.033 (0.84)  Sentence2: 4.033 (0.84) | |
|  | 强森在攀岩时看到了一只猣。  Johnson saw a ZONG while climbing. | 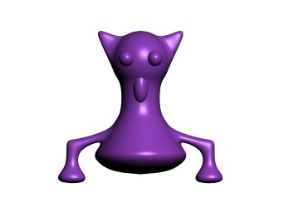 | | ZONG living in the mountains | |  |  |
| 猡  LUO | 邦妮在池塘边看到一只猡。  Bonnie saw a LUO by the pond. | 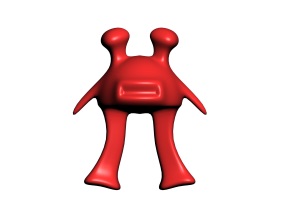 | | Living LUO | | Name: 4.433 (0.67)  object: 4.233 (0.76)  state1: 4.100 (0.83)  state2: 4.333 (0.83)  Sentence1: 4.167 (0.82)  Sentence2: 4.100 (0.75) | |
|  | 邦妮在锅里看到一只猡。  Bonnie saw a LUO in the pot. | 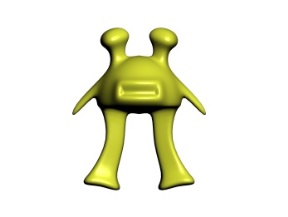 | | Dead LUO | |  |  |
| 猇  XIAO | 达茜在襁褓里看到一只猇。  Darcy saw a XIAO in the swaddle. | 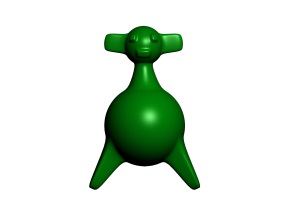 | | Young XIAO | | Name: 4.333 (0.65)  object: 4.667 (0.54)  state1: 4.200 (0.83)  state2: 4.100 (0.70)  Sentence1: 4.400 (0.71)  Sentence2: 4.200 (0.65) | |
|  | 达茜看到一只猇步履蹒跚。  Darcy saw a XIAO walking hobblely. | 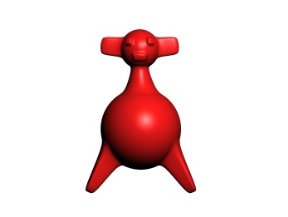 | | Old XIAO | |  |  |
| 狷  JUAN | 大卫看到一匹狷在散步。  David saw a JUAN strolling. | 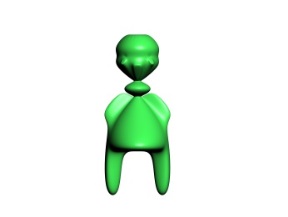 | | JUAN at leisure | | Name: 4.133 (0.81)  object: 4.367 (0.75)  state1: 4.600 (0.71)  state2: 4.067 (0.81)  Sentence1: 4.067 (0.81)  Sentence2: 4.033 (0.80) | |
|  | 大卫看到一匹狷在狂奔。  David saw a JUAN galloping. | 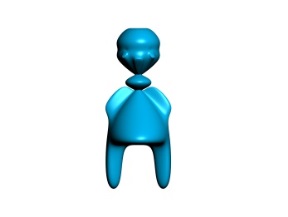 | | JUAN when nervous | |  |  |
| 萱  XUAN | 约翰吐出了难吃的萱。  John spit out the unpalatable XUAN. | 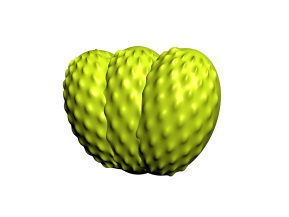 | | Astringent XUAN | | Name: 4.067 (0.77)  object: 4.400 (0.84)  state1: 4.167 (0.82)  state2: 4.200 (0.75)  Sentence1: 4.133 (0.72)  Sentence2: 4.033 (0.84) | |
|  | 约翰咬了口好吃的萱。  John took a bite of the delicious XUAN. | 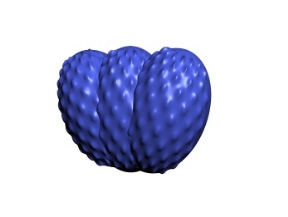 | | Delicious XUAN | |  |  |
| 萖  WAN | 威廉给萖浇水。  William watered the WAN. | 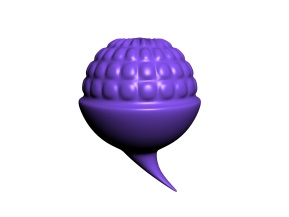 | | Growing WAN | | Name: 4.167 (0.90)  object: 4.067 (0.77)  state1: 4.300 (0.90)  state2: 4.333 (0.70)  Sentence1: 4.033 (0.87)  Sentence2: 4.333 (0.70) | |
|  | 威廉摘下了萖。  William picked off the WAN. | 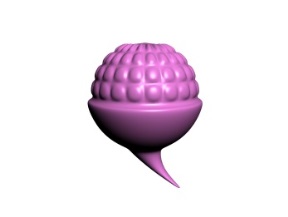 | | Ripe WAN | |  |  |
| 茠  HAO | 唐纳在窗台上看到了一盆茠。  Donner saw a pot of HAO on the windowsill. | 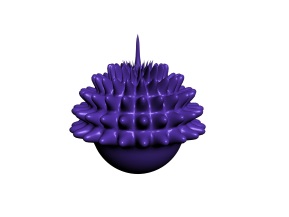 | | HAO growing indoors | | Name: 4.100 (0.83)  object: 4.333 (0.79)  state1: 4.467 (0.81)  state2: 4.033 (0.91)  Sentence1: 4.200 (0.79)  Sentence2: 4.200 (0.70) | |
|  | 唐纳透过车窗看到了一丛茠。  Donner saw a bunch of HAO through the car window. | 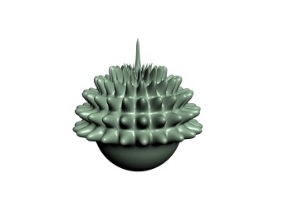 | | HAO growing in wild | |  |  |
| 苚  YONG | 约翰从果篮中挑出一颗苚丢掉。  John threw away a YONG from the fruit basket. | 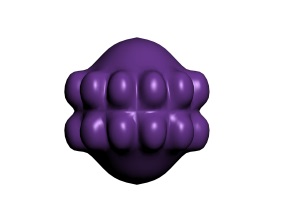 | | Dry YONG | | Name: 4.133 (0.81)  object: 4.133 (0.88)  state1: 4.033 (0.84)  state2: 4.033 (0.80)  Sentence1: 4.167 (0.86)  Sentence2: 4.167 (0.78) | |
|  | 约翰将一颗苚放进果篮。  John put a YONG into the fruit basket. | 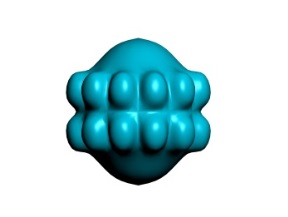 | | Full YONG | |  |  |
| 獬  XIE | 杰克看到一只慵懒的獬。Jack saw a lazy XIE. | | 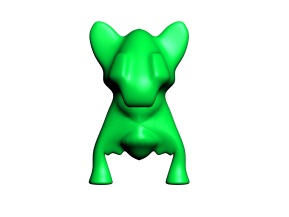 | | Satiate XIE | | Name: 4.100 (0.83)  object: 4.567 (0.76)  state1: 4.700 (0.64)  state2: 4.033 (0.80)  Sentence1: 4.200 (0.91)  Sentence2: 4.267 (0.77) |
|  | 杰克看到一只捕猎的獬。  Jack saw a hunting XIE. | | 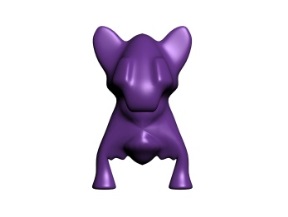 | | Hungry XIE | |  |
| 狺  YIN | 伊万看到了一匹健康的狺。  Ivan saw a healthy YIN. | | 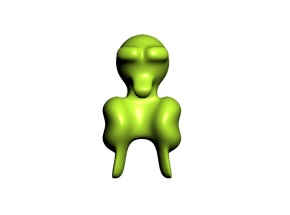 | | Healthy YIN | | Name: 4.133 (0.81)  object: 4.267 (0.68)  state1: 4.833 (0.52)  state2: 4.633 (0.48)  Sentence1: 4.100 (0.83)  Sentence2: 4.033 (0.75) |
|  | 伊万看到了一匹生病的狺。  Ivan saw a sick YIN. | | 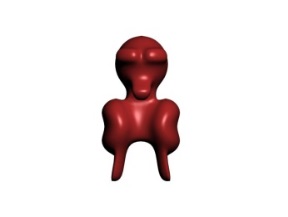 | | Sick YIN | |  |
| 猘  ZHI | 皮特看到一只蹦跳的猘。  Pete saw a jumping ZHI. | | 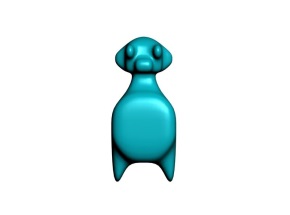 | | Pleasant ZHI | | Name: 4.000 (0.73)  object: 4.233 (0.84)  state1: 4.067 (0.85)  state2: 4.400 (0.71)  Sentence1: 4.167 (0.82)  Sentence2: 4.067 (0.85) |
|  | 皮特看到一只低吼的猘。  Peter saw a growling ZHI. | | 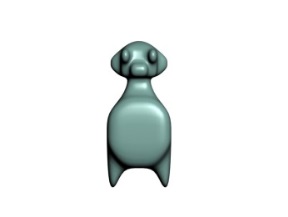 | | Angry ZHI | |  |
| 獲  HUO | 黛比看到一只獲在求偶。  Debbie saw a courting HUO. | | 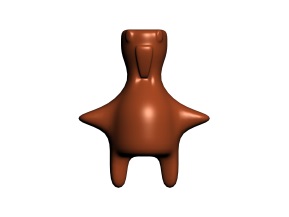 | | Male HUO | | Name: 4.100 (0.79)  object: 4.933 (0.36)  state1: 4.733 (0.63)  state2: 4.067 (0.93)  Sentence1: 4.100 (0.83)  Sentence2: 4.233 (0.76) |
|  | 黛比看到一只獲在孵蛋。  Debbie saw a brooding HUO. | | 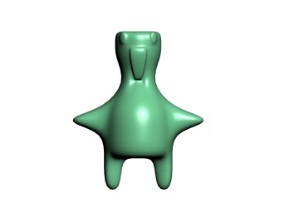 | | Femal HUO | |  |
| 葔  HOU | 露西在路边的雪堆中看到一根葔。  Lucy saw a HOU in the snowdrift on the side of the road. | | 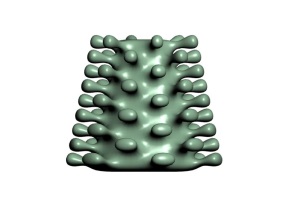 | | HOU in cold weather | | Name: 4.167 (0.78)  object: 4.567 (0.72)  state1: 4.700 (0.69)  state2: 4.233 (0.88)  Sentence1: 4.167 (0.78)  Sentence2: 4.167 (0.86) |
|  | 露西在路边的花丛中看到一根葔。  Lucy saw a HOU among the flowers on the side of the road. | | 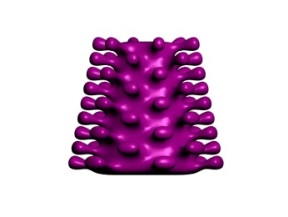 | | HOU in warm weather | |  |
| 葄  ZUO | 艾比看到一串含苞待放的葄。  Abby saw a budding ZUO. | | 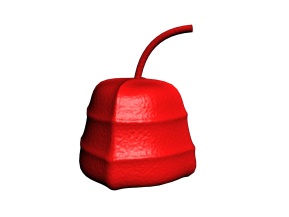 | | ZUO in spring | | Name: 4.133 (0.81)  object: 4.267 (0.81)  state1: 4.733 (0.68)  state2: 4.100 (0.79)  Sentence1: 4.200 (0.83)  Sentence2: 4.067 (0.77) |
|  | 艾比看到一串硕果累累的葄。  Abby saw a fruitful ZUO. | | 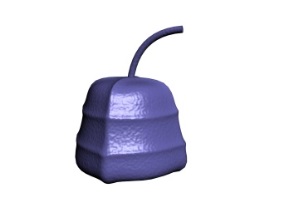 | | ZUO in autumn | |  |
| 蒍  WEI | 萨尔在潜水时摘下一颗蒍。  Thrall took off a WEI while diving. | | 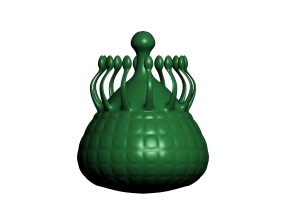 | | WEI growing in water | | Name: 4.100 (0.83)  object: 4.800 (0.48)  state1: 4.667 (0.70)  state2: 4.167 (0.82)  Sentence1: 4.100 (0.83)  Sentence2: 4.167 (0.82) |
|  | 萨尔在远足时找到一颗蒍。  Thrall found a WEI while hiking. | | 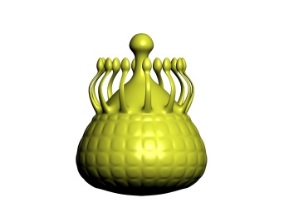 | | WEI growing on land | |  |

**Pre-testing data for Experiment 2**

| Name | Senences | | Objects | Score (M, SD) |
| --- | --- | --- | --- | --- |
|  | **Clear** | **Unclear** |  |  |
| 猣  ZONG | 强森在陡峭的山崖上看到了一只猣。  Johnson saw a ZONG on the steep cliff. | 强森在攀岩时看到了一只猣。  Johnson saw a ZONG while climbing. | 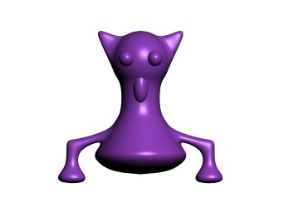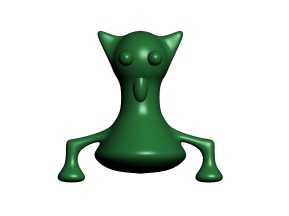 | clear1: 3.733 (0.85)  clear2: 3.500 (0.62)  unclear1: 1.767 (0.76)  unclear2: 1.833 (0.86) |
|  | 强森在宽阔的草地上看到了一只猣。  Johnson saw a ZONG on the vast grassland. | 强森在放牧时看到了一只猣。  Johnson saw a ZONG while grazing. |  |  |
| 猡  LUO | 邦妮在锅里看到一只煮熟的猡。  Bonnie saw a cooked LUO in the pot. | 邦妮在锅里看到一只猡。  Bonnie saw a LUO in the pot. | 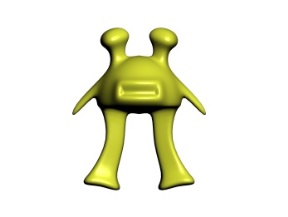  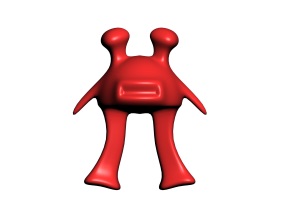 | clear1: 3.767 (0.72)  clear2: 3.667 (0.83) unclear1: 1.900 (0.83)  unclear2: 1.733 (0.81) |
|  | 邦妮在池塘边看到一只蹦跳的猡。  Bonnie saw a jumping LUO by the pond. | 邦妮在池塘边看到一只猡。  Bonnie saw a LUO by the pond. |  |  |
| 猇  XIAO | 达茜看到一只老迈的猇。  Darcy saw an elderly XIAO walking hobblely. | 达茜看到一只猇步履蹒跚。  Darcy saw a XIAO walking hobblely. | 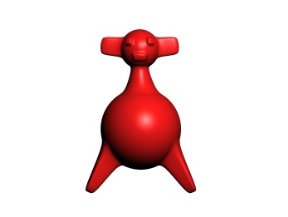  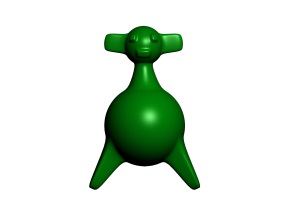 | clear1: 3.967 (0.75)  clear2: 3.567 (0.76)  unclear1: 1.700 (0.86)  unclear2: 1.967 (0.75) |
|  | 达茜在看到一只幼小的猇。  Darcy saw a young XIAO in the swaddle. | 达茜在襁褓里看到一只猇。  Darcy saw a XIAO in the swaddle. |  |  |
| 狷  JUAN | 大卫看到一匹狷慌乱的狂奔。  David saw a JUAN galloping wildly in panic. | 大卫看到一匹狷在狂奔。  David saw a JUAN galloping. | 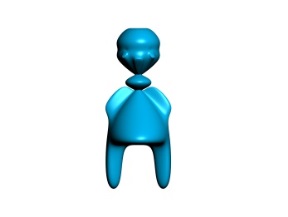  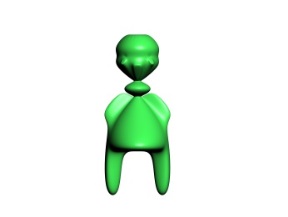 | clear1: 3.833 (0.73)  clear2: 3.467 (0.67)  unclear1: 1.967 (0.80)  unclear2: 1.633 (0.75) |
|  | 大卫看到一匹狷闲适的散步。  David saw a JUAN strolling leisurely. | 大卫看到一匹狷在散步。  David saw a JUAN strolling. |  |  |
| 萱  XUAN | 约翰咬了口好吃的萱。  John took a bite of the delicious XUAN. | 约翰吞下了萱。  John gulped the XUAN. | 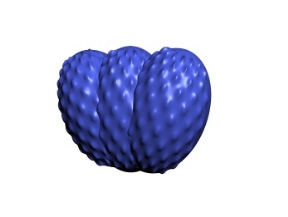  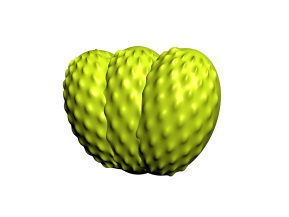 | clear1: 4.367 (0.80)  clear2: 4.000 (0.89)  unclear1: 2.000 (0.82)  unclear2: 1.767 (0.84) |
|  | 约翰吐出了难吃的萱。  John spit out the unpalatable XUAN. | 约翰吐出了萱。  John spit out the XUAN. |  |  |
| 萖  WAN | 威廉将熟透了的萖摘了下来。  William picked off the mature WAN. | 威廉摘下了萖。  William picked off the WAN. | 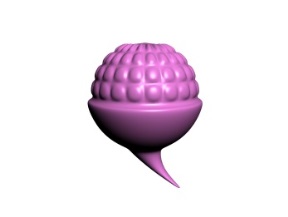  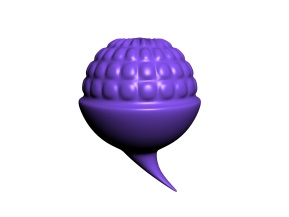 | clear1: 3.567 (0.80)  clear2: 3.533 (0.62)  unclear1: 2.033 (0.87)  unclear2: 2.267 (0.77) |
|  | 威廉为茁壮成长的萖浇水。  William watered the thriving WAN. | 威廉给萖浇水。  William watered the WAN. |  |  |
| 茠  HAO | 唐纳在山坡上看到了一丛茠。  Donner saw a bunch of HAO on the hillside. | 唐纳透过车窗看到了一丛茠。  Donner saw a bunch of HAO through the car window. | 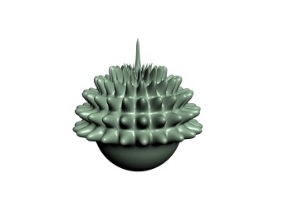  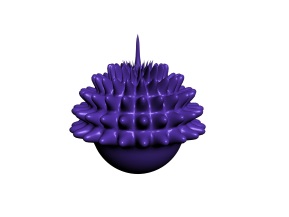 | clear1: 3.500 (0.67)  clear2: 3.700 (0.82)  unclear1: 1.767 (0.67)  unclear2: 2.133 (0.81) |
|  | 唐纳在屋里看到了一盆茠。  Donner saw a pot of HAO in the room. | 唐纳在窗台上看到了一盆茠。  Donner saw a pot of HAO on the windowsill. |  |  |
| 苚  YONG | 约翰拾起了一颗圆润的苚。  John picked up a fruity YONG. | 约翰将一颗苚放进果篮。  John put a YONG into the fruit basket. | 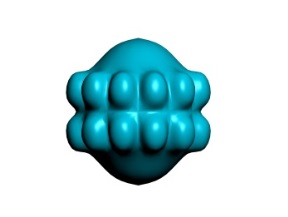  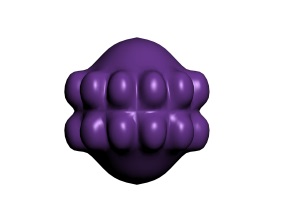 | clear1: 4.167 (0.82)  clear2: 3.733 (0.81)  unclear1: 1.900 (0.75)  unclear2: 2.000 (0.82) |
|  | 约翰丢掉了一颗干瘪的苚。  John threw away a shriveled YONG. | 约翰从果篮中挑出一颗苚丢掉。  John threw away a YONG from the fruit basket. |  |  |
| 獬  XIE | 杰克看到一只獬打着饱嗝。  Jack saw a Xie burping heavily. | 杰克看到一只慵懒的獬。 Jack saw a lazy XIE. | 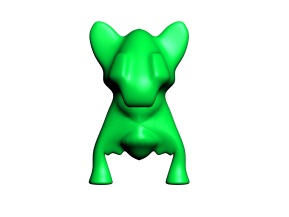  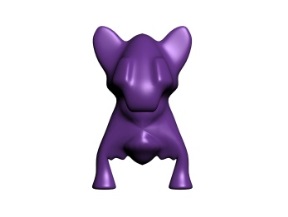 | clear1: 3.567 (0.72)  clear2: 3.867 (0.88)  unclear1: 1.733 (0.81)  unclear2: 2.200 (0.75) |
|  | 杰克看到一只獬正在进食。  Jack saw an eating XIE. | 杰克看到一只捕猎的獬。Jack saw a hunting XIE. |  |  |
| 狺  YIN | 伊万看到了一匹生病的狺。  Ivan saw a sick YIN. | 伊万看到了一匹无精打采的狺。  Ivan saw a listless YIN. | 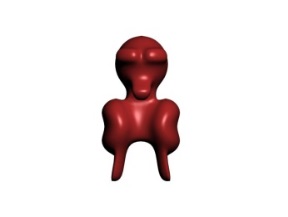  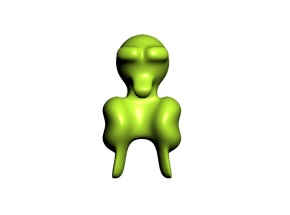 | clear1: 3.867 (0.92)  clear2: 3.800 (0.83)  unclear1: 2.000 (0.82)  unclear2: 2.033 (0.75) |
|  | 伊万看到了一匹健康的狺。  Ivan saw a healthy YIN. | 伊万看到了一匹精神抖擞的狺。  Ivan saw a vigorous YIN. |  |  |
| 猘  ZHI | 皮特看到一只愤怒的猘。  Peter saw an angery ZHI. | 皮特看到一只低吼的猘。  Peter saw a growling ZHI. | 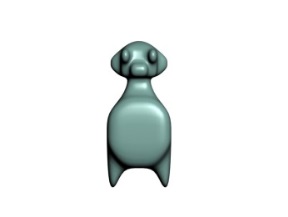  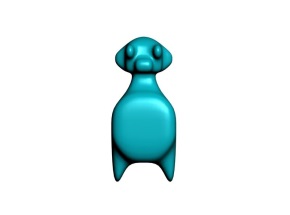 | clear1: 4.067 (0.85)  clear2: 4.100 (0.87)  unclear1: 2.133 (0.81)  unclear2: 2.133 (0.62) |
|  | 皮特看到一只高兴的猘。  Peter saw a happy ZHI. | 皮特看到一只蹦跳的猘。  Peter saw a jumping ZHI. |  |  |
| 獲  HUO | 黛比看到一只獲在孵蛋。  Debbie saw a brooding HUO. | 黛比看到一只獲在为幼崽觅食。  Debbie saw a HUO foraging for the cub. | 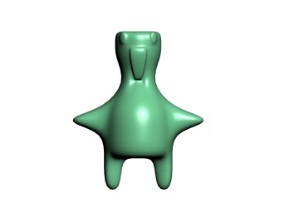  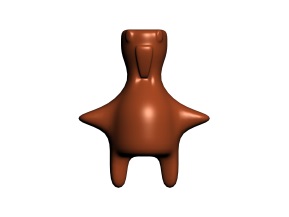 | clear1: 4.167 (0.78)  clear2: 3.900 (0.87)  unclear1: 1.933 (0.81)  unclear2: 2.100 (0.79) |
|  | 黛比看到一只獲在求偶。  Debbie saw a courting HUO. | 黛比看到一只獲在保护领地。  Debbie saw a HUO protecting territory. |  |  |
| 葔  HOU | 露西看到一根葔在暖风中摇摆。  Lucy saw a HOU swaying in the warm wind. | 露西在路边的花丛中看到一根葔。  Lucy saw a HOU among the flowers on the side of the road. | 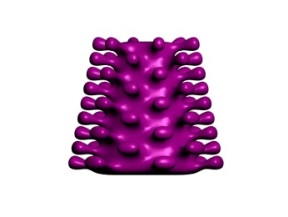  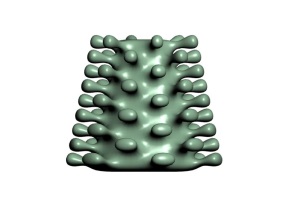 | clear1: 3.833 (0.86)  clear2: 3.700 (0.74)    unclear1: 1.833 (0.86)  unclear2: 1.667 (0.75) |
|  | 露西看到一根葔在寒风中摇曳。  Lucy saw a HOU swaying in the cold wind. | 露西在路边的雪堆中看到一根葔。  Lucy saw a HOU in the snowdrift on the side of the road. |  |  |
| 葄  ZUO | 艾比在清爽的秋风里看到一串葄。  Abby saw a ZUO in the refreshing autumn breeze. | 艾比看到一串硕果累累的葄。  Abby saw a fruitful ZUO. | 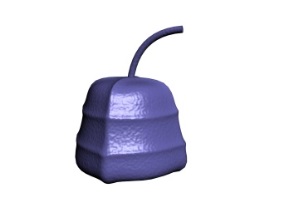  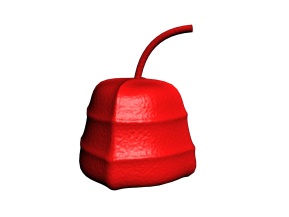 | clear1: 4.200 (0.87)  clear2: 3.633 (0.71)  unclear1: 2.067 (0.77)  unclear2: 1.867 (0.72) |
|  | 艾比在和煦的春风里看到一串葄。  Abby saw a ZUO in the warm spring breeze. | 艾比看到一串含苞待放的葄。  Abby saw a budding ZUO. |  |  |
| 蒍  WEI | 萨尔从泥土里挖出一颗蒍。  Thrall dug out a WEI from the soil. | 萨尔在远足时找到一颗蒍。  Thrall found a WEI while hiking. | 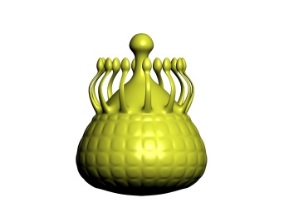  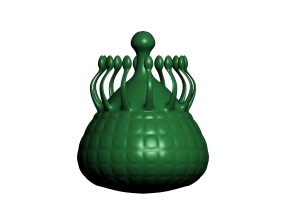 | clear1: 3.900 (0.87) clear2: 3.933 (0.85)  unclear1: 2.167 (0.82)  unclear2: 1.900 (0.87) |
|  | 萨尔从水里捞上来一颗蒍。  Thrall picked up a WEI from the water. | 萨尔在潜水时摘下一颗蒍。  Thrall took off a WEI while diving. |  |  |

**Material novelty evaluation for Experiment 1**

**材料新颖度测评**

欢迎参加我们的实验，请对下面的实验材料打分。其中，图片的新颖程度和名字的新颖程度打分范围为1~5分(分值越高代表越新颖)；名字是否能联想到颜色(是或否)。

Welcome to participate in our experiment, please rate the following experimental materials. Among them, the score range of the novelty of the picture and the novelty of the name is 1-5 points (the higher the score, the more novel); whether the name is associated with a color (yes or no).

Chinese and English comparison table of questions

| 图片的新颖程度 | The novelty of the picture |
| --- | --- |
| 名字的新颖程度 | The novelty of the name |
| 名字是否能联想到颜色 | Whether the name is associated with a color |


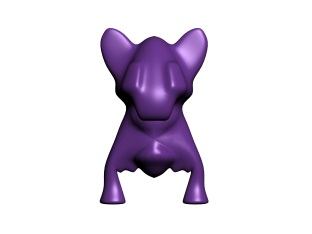

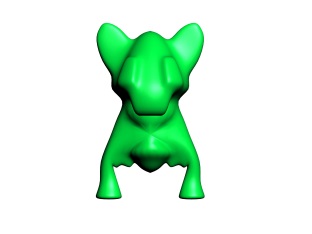

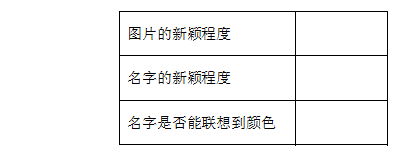


獬(Xie)


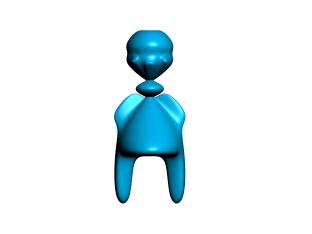

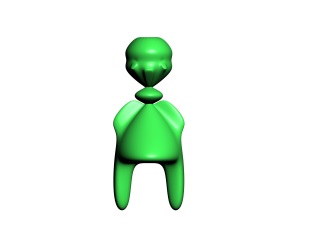

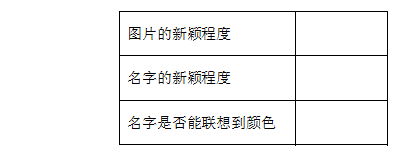


狷(Juan)


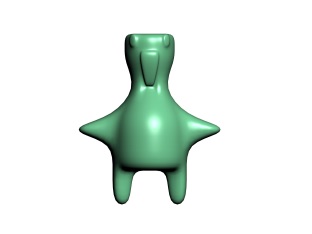

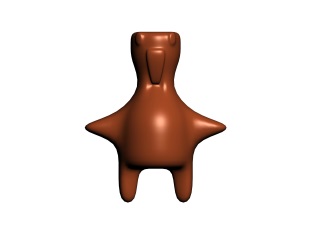

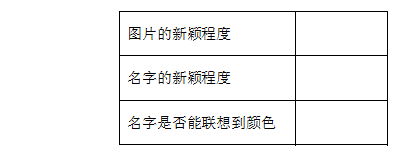


獲(Huo)


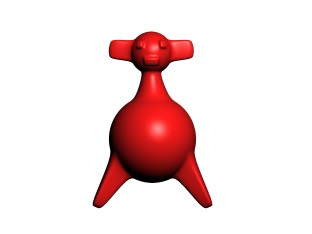

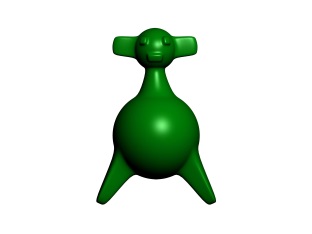

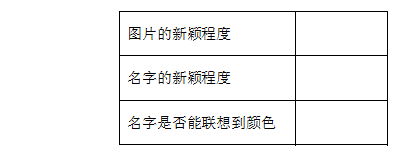


猇(Xiao)


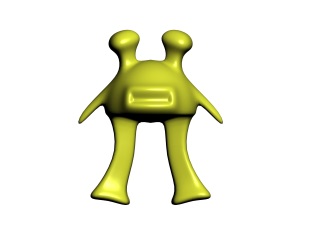

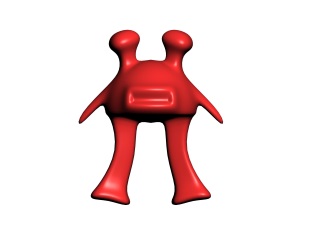

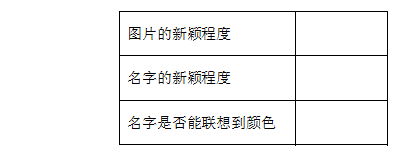


猡(Luo)


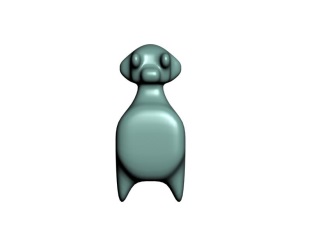

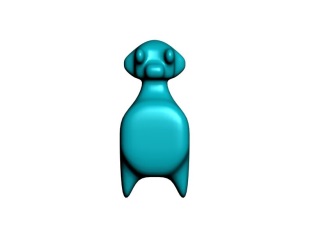

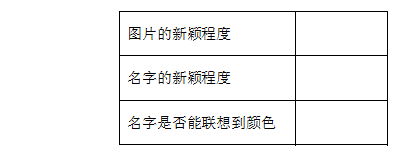


猘(Zhi)


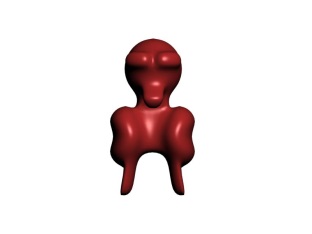

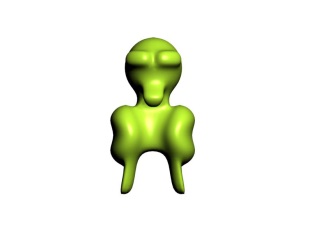

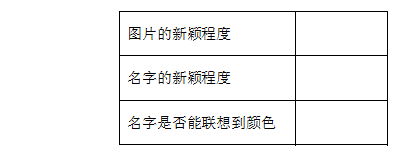


狺(Yin)


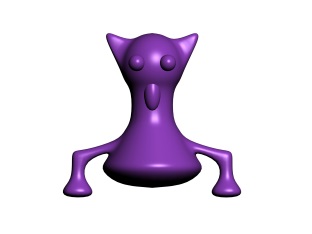

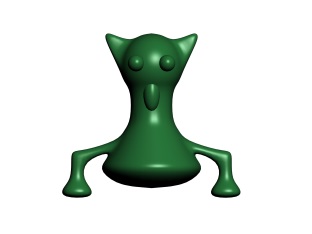

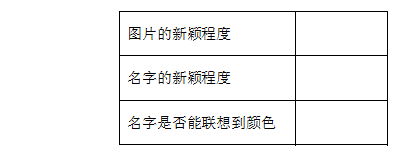


猣(Zong)


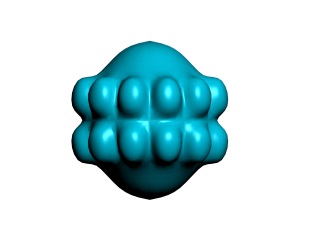

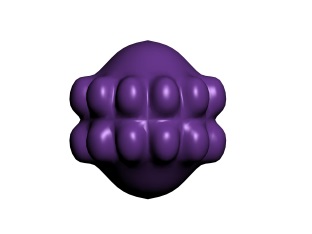

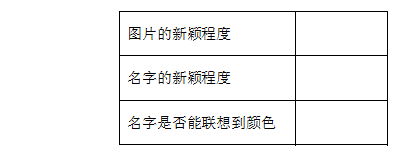


苚(Yong)


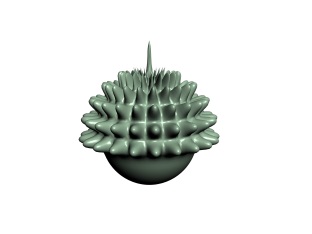

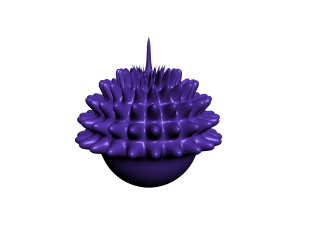

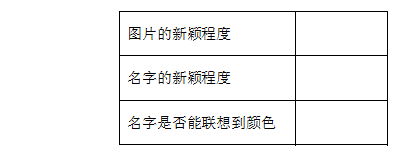


茠(Hao)


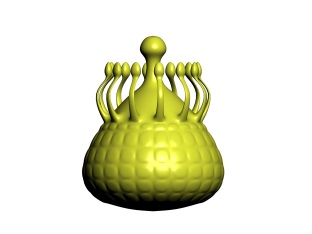

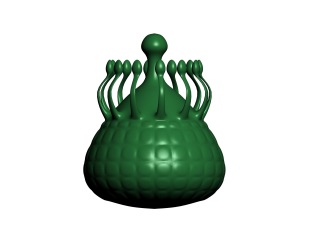

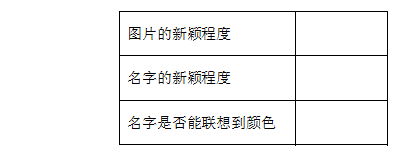


蒍(Wei)


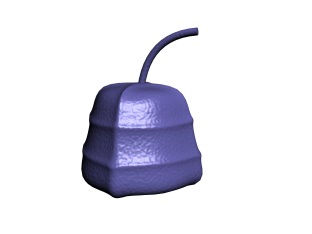

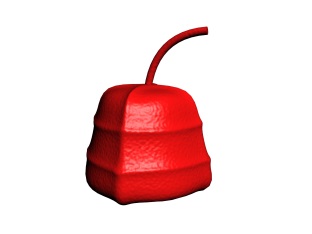

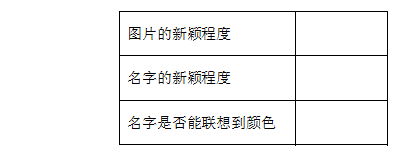


葄(Zuo)


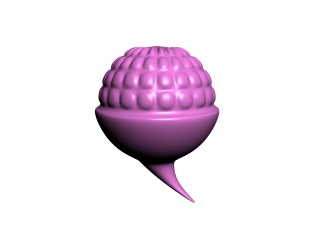

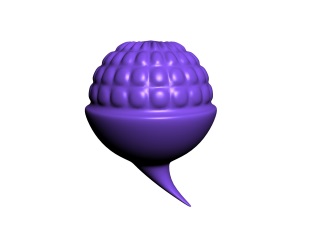

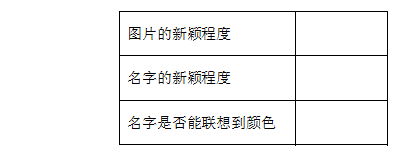


萖(Wan)


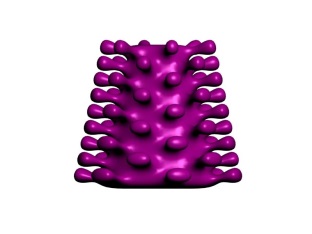


葔(Hou)

萱(Xuan)

**Object description and corresponding sentence evaluation for Experiment 1**

**物体描述及对应句子测评**

欢迎参加我们的实验，请对下面的实验材料打分。状态描述意味着是否清楚地理解了所描述物体的状态，1~5分(分值越高代表越理解)；句子理解意味着能否清楚地理解句子所描述的信息，1~5分(分值越高代表越理解)。

Welcome to participate in our experiment, please rate the following experimental materials. State description means whether the state of the described object is clearly understood on a scale of 1 to 5 (the higher the score, the better the understanding); Sentence comprehension means that you can clearly understand the information described in the sentence, on a scale of 1 to 5 (the higher the score, the better the understanding).

獬(Xie)

| 吃饱的獬  Satiate XIE |  | 饥饿的獬  Hungry XIE |  |
| --- | --- | --- | --- |
| 杰克看到一只慵懒的獬。  Jack saw a lazy XIE. |  | 杰克看到一只捕猎的獬。  Jack saw a hunting XIE. |  |

狷(Juan)

| 紧张时的狷  JUAN when nervous |  | 悠闲时的狷  JUAN at leisure |  |
| --- | --- | --- | --- |
| 大卫看到一匹狷在狂奔。  David saw a JUAN  galloping. |  | 大卫看到一匹狷在散步。  David saw a JUAN  strolling. |  |

獲(Huo)

| 雌性的獲  Femal HUO |  | 雄性的獲  Male HUO |  |
| --- | --- | --- | --- |
| 黛比看到一只獲在孵蛋。  Debbie saw a brooding  HUO. |  | 黛比看到一只獲在求偶。  Debbie saw a courting  HUO. |  |

猇(Xiao)

| 老年期的猇  Old XIAO |  | 幼年期的猇  Young XIAO |  |
| --- | --- | --- | --- |
| 达茜看到一只猇步履蹒跚。  Darcy saw a XIAO  walking hobblely. |  | 达茜在襁褓里看到一只猇。  Darcy saw a XIAO in the  swaddle. |  |

猡(Luo)

| 死掉的猡  Dead LUO |  | 活着的猡  Living LUO |  |
| --- | --- | --- | --- |
| 邦妮在锅里看到一只猡。  Bonnie saw a LUO in the  pot. |  | 邦妮在池塘边看到一只猡。  Bonnie saw a LUO by the  pond. |  |

猘(Zhi)

| 愤怒时的猘  Angry ZHI |  | 愉快时的猘  Pleasant ZHI |  |
| --- | --- | --- | --- |
| 皮特看到一只低吼的猘。  Peter saw a growling ZHI. |  | 皮特看到一只蹦跳的猘。  Peter saw a jumping ZHI. |  |

狺(Yin)

| 生病的狺  Sick YIN |  | 健康的狺  Healthy YIN |  |
| --- | --- | --- | --- |
| 伊万看到了一匹生病的狺。  Ivan saw a sick YIN. |  | 伊万看到了一匹健康的狺。  Ivan saw a healthy YIN. |  |

猣(Zong)

| 生活在山区的猣  ZONG living in the mountains |  | 生活在草原的猣  ZONG living in the grassland |  |
| --- | --- | --- | --- |
| 强森在攀岩时看到了一只猣。  Johnson saw a ZONG while climbing. |  | 强森在放牧时看到了一只猣。  Johnson saw a ZONG while grazing. |  |

苚(Yong)

| 饱满时的苚  Full YONG |  | 干枯时的苚  Dry YONG |  |
| --- | --- | --- | --- |
| 约翰将一颗苚放进果篮。  John put a YONG into the fruit basket. |  | 约翰从果篮中挑出一颗苚丢掉。  John threw away a YONG from the fruit basket. |  |

茠(Hao)

| 野外生长的茠  HAO growing in wild |  | 室内生长的茠  HAO growing indoors |  |
| --- | --- | --- | --- |
| 唐纳透过车窗看到了一丛茠。  Donner saw a bunch of HAO through the car window. |  | 唐纳在窗台上看到了一盆茠。  Donner saw a pot of HAO on the windowsill. |  |

蒍(Wei)

| 生长在土中的蒍  WEI growing on land |  | 生长在水中的蒍  WEI growing in water |  |
| --- | --- | --- | --- |
| 萨尔在远足时找到一颗蒍。  Thrall found a WEI while hiking. |  | 萨尔在潜水时摘下一颗蒍。  Thrall took off a WEI while diving. |  |

葄(Zuo)

| 秋天时的葄  ZUO in autumn |  | 春天时的葄  ZUO in spring |  |
| --- | --- | --- | --- |
| 艾比看到一串硕果累累的葄。  Abby saw a fruitful ZUO. |  | 艾比看到一串含苞待放的葄。  Abby saw a budding ZUO. |  |

萖(Wan)

| 成熟的萖  Ripe WAN |  | 生长的萖  Growing WAN |  |
| --- | --- | --- | --- |
| 威廉摘下了萖。  William picked off the WAN. |  | 威廉给萖浇水。  William watered the WAN. |  |

葔(Hou)

| 温暖气候中的葔  HOU in warm weather |  | 寒冷气候中的葔  HOU in cold weather |  |
| --- | --- | --- | --- |
| 露西在路边的花丛中看到一根葔。  Lucy saw a HOU in the snowdrift on the side of the road. |  | 露西在路边的雪堆中看到一根葔。  Lucy saw a HOU among the flowers on the side of the road. |  |

萱(Xuan)

| 美味的萱  Delicious XUAN |  | 生涩的萱  Astringent XUAN |  |
| --- | --- | --- | --- |
| 约翰咬了口好吃的萱。  John took a bite of the delicious XUAN. |  | 约翰吐出了难吃的萱。  John spit out the unpalatable XUAN. |  |

**Object description and corresponding sentence evaluation for Experiment 2**

**材料描述及对应句子测评**

欢迎参加我们的实验。每种物体下面的第一行描述了物体的状态，对下面所对应的两个句子。请进行1~5分的评分，句子越能描述物体的状态则评分越高，反之评分越低。

Welcome to our experiment. The first line below each object describes the state of the object, and the corresponding two sentences. Please score on a scale of 1 to 5. The more the sentence describes the state of the object, the higher the score is.

獬(Xie)

| 吃饱的獬  Satiate XIE | | 饥饿的獬  Hungry XIE | |
| --- | --- | --- | --- |
| 杰克看到一只慵懒的獬。  Jack saw a lazy XIE. |  | 杰克看到一只捕猎的獬。  Jack saw a hunting XIE. |  |
| 杰克看到一只獬打着饱嗝。  Jack saw a Xie burping heavily. |  | 杰克看到一只獬正在进食。  Jack saw an eating XIE. |  |

狷(Juan)

| 紧张时的狷  JUAN when nervous | | 悠闲时的狷  JUAN at leisure | |
| --- | --- | --- | --- |
| 大卫看到一匹狷在狂奔。  David saw a JUAN galloping. |  | 大卫看到一匹狷在散步。  David saw a JUAN strolling. |  |
| 大卫看到一匹狷慌乱的狂奔。  David saw a JUAN galloping wildly in panic. |  | 大卫看到一匹狷闲适的散步。  David saw a JUAN strolling leisurely. |  |

獲(Huo)

| 雌性的獲  Femal HUO | | 雄性的獲  Male HUO | |
| --- | --- | --- | --- |
| 黛比看到一只獲在为幼崽觅食。  Debbie saw a HUO foraging for the cub. |  | 黛比看到一只獲在保护领地。  Debbie saw a HUO protecting territory. |  |
| 黛比看到一只獲在孵蛋。  Debbie saw a brooding HUO. |  | 黛比看到一只獲在求偶。  Debbie saw a courting HUO. |  |

猇(Xiao)

| 老年期的猇  Old XIAO | | 幼年期的猇  Young XIAO | |
| --- | --- | --- | --- |
| 达茜看到一只猇步履蹒跚。  Darcy saw a XIAO walking hobblely. |  | 达茜在襁褓里看到一只猇。  Darcy saw a XIAO in the swaddle. |  |
| 达茜看到一只老迈的猇。  Darcy saw an elderly XIAO walking hobblely. |  | 达茜看到一只幼小的猇。  Darcy saw a young XIAO in the swaddle. |  |

猡(Luo)

| 死掉的猡  Dead LUO | | 活着的猡  Living LUO | |
| --- | --- | --- | --- |
| 邦妮在锅里看到一只猡。  Bonnie saw a LUO in the pot. |  | 邦妮在池塘边看到一只猡。  Bonnie saw a LUO by the pond. |  |
| 邦妮在锅里看到一只煮熟的猡。  Bonnie saw a cooked LUO in the pot. |  | 邦妮在池塘边看到一只蹦跳的猡。  Bonnie saw a jumping LUO by the pond. |  |

猘(Zhi)

| 愤怒时的猘  Angry ZHI | | 愉快时的猘  Pleasant ZHI | |
| --- | --- | --- | --- |
| 皮特看到一只低吼的猘。  Peter saw a growling ZHI. |  | 皮特看到一只蹦跳的猘。  Peter saw a jumping ZHI. |  |
| 皮特看到一只愤怒的猘。  Peter saw an angery ZHI. |  | 皮特看到一只高兴的猘。  Peter saw a happy ZHI. |  |

狺(Yin)

| 生病的狺  Sick YIN | | 健康的狺  Healthy YIN | |
| --- | --- | --- | --- |
| 伊万看到了一匹无精打采的狺。  Ivan saw a listless YIN. |  | 伊万看到了一匹精神抖擞的狺。  Ivan saw a vigorous YIN. |  |
| 伊万看到了一匹生病的狺。  Ivan saw a sick YIN. |  | 伊万看到了一匹健康的狺。  Ivan saw a healthy YIN. |  |

猣(Zong)

| 生活在山区的猣  ZONG living in the mountains | | 生活在草原的猣  ZONG living in the grassland | |
| --- | --- | --- | --- |
| 强森在攀岩时看到了一只猣。  Johnson saw a ZONG while climbing. |  | 强森在放牧时看到了一只猣。  Johnson saw a ZONG while grazing. |  |
| 强森在陡峭的山崖上看到了一只猣。  Johnson saw a ZONG on the steep cliff. |  | 强森在宽阔的草地上看到了一只猣。  Johnson saw a ZONG on the vast grassland. |  |

苚(Yong)

| 饱满时的苚  Full YONG | | 干枯时的苚  Dry YONG | |
| --- | --- | --- | --- |
| 约翰将一颗苚放进果篮。  John put a YONG into the fruit basket. |  | 约翰从果篮中挑出一颗苚丢掉。  John threw away a YONG from the fruit basket. |  |
| 约翰拾起了一颗圆润的苚。  John picked up a fruity YONG. |  | 约翰丢掉了一颗干瘪的苚。  John threw away a shriveled YONG. |  |

茠(Hao)

| 野外生长的茠  HAO growing in wild | | | | 室内生长的茠  HAO growing indoors | | | |
| --- | --- | --- | --- | --- | --- | --- | --- |
| 唐纳透过车窗看到了一丛茠。  Donner saw a bunch of HAO through the car window. | |  | | 唐纳在窗台上看到了一盆茠。  Donner saw a pot of HAO on the windowsill. | |  | |
| 唐纳在山坡上看到了一丛茠。  Donner saw a bunch of HAO on the hillside. | |  | | 唐纳在屋里看到了一盆茠。  Donner saw a pot of HAO in the room. | |  | |

蒍(Wei)

| 生长在土中的蒍  WEI growing on land | | 生长在水中的蒍  WEI growing in water | |
| --- | --- | --- | --- |
| 萨尔在远足时找到一颗蒍。  Thrall found a WEI while hiking. |  | 萨尔在潜水时摘下一颗蒍。  Thrall took off a WEI while diving. |  |
| 萨尔从泥土里挖出一颗蒍。  Thrall dug out a WEI from the soil. |  | 萨尔从水里捞上来一颗蒍。  Thrall picked up a WEI from the water. |  |

葄(Zuo)

| 秋天时的葄  ZUO in autumn | | 春天时的葄  ZUO in spring | |
| --- | --- | --- | --- |
| 艾比看到一串硕果累累的葄。  Abby saw a fruitful ZUO. |  | 艾比看到一串含苞待放的葄。  Abby saw a budding ZUO. |  |
| 艾比在清爽的秋风里看到一串葄。  Abby saw a ZUO in the refreshing autumn breeze. |  | 艾比在和煦的春风里看到一串葄。  Abby saw a ZUO in the warm spring breeze. |  |

萖(Wan)

| 成熟的萖  Ripe WAN | | 生长的萖  Growing WAN | |
| --- | --- | --- | --- |
| 威廉摘下了萖。  William picked off the WAN. |  | 威廉给萖浇水。  William watered the WAN. |  |
| 威廉将熟透了的萖摘了下来。  William picked off the mature WAN. |  | 威廉为茁壮成长的萖浇水。  William watered the thriving WAN. |  |

葔(Hou)

| 温暖气候中的葔  HOU in warm weather | | 寒冷气候中的葔  HOU in cold weather | |
| --- | --- | --- | --- |
| 露西在路边的花丛中看到一根葔。  Lucy saw a HOU among the flowers on the side of the road. |  | 露西在路边的雪堆中看到一根葔。  Lucy saw a HOU in the snowdrift on the side of the road. |  |
| 露西看到一根葔在暖风中摇摆。  Lucy saw a HOU swaying in the warm wind. |  | 露西看到一根葔在寒风中摇曳。  Lucy saw a HOU swaying in the cold wind. |  |

萱(Xuan)

| 美味的萱  Delicious XUAN | | 生涩的萱  Astringent XUAN | |
| --- | --- | --- | --- |
| 约翰吞下了萱。  John gulped the XUAN. |  | 约翰吐出了萱。  John spit out the XUAN. |  |
| 约翰咬了口好吃的萱。  John took a bite of the delicious XUAN. |  | 约翰吐出了难吃的萱。  John spit out the unpalatable XUAN. |  |
